# Multidrug-Resistant and Extended Spectrum Beta-Lactamase-Producing *Escherichia coli* in Dutch Surface Water and Wastewater

**DOI:** 10.1371/journal.pone.0127752

**Published:** 2015-06-01

**Authors:** Hetty Blaak, Gretta Lynch, Ronald Italiaander, Raditijo A. Hamidjaja, Franciska M. Schets, Ana Maria de Roda Husman

**Affiliations:** 1 Centre for Zoonoses and Environmental Microbiology, National Institute for Public Health and the Environment (RIVM), Bilthoven, the Netherlands; 2 Institute for Risk Assessment Sciences, Utrecht University, Utrecht, the Netherlands; St. Petersburg Pasteur Institute, RUSSIAN FEDERATION

## Abstract

**Objective:**

The goal of the current study was to gain insight into the prevalence and concentrations of antimicrobial resistant (AMR) *Escherichia coli* in Dutch surface water, and to explore the role of wastewater as AMR contamination source.

**Methods:**

The prevalence of AMR *E*. *coli* was determined in 113 surface water samples obtained from 30 different water bodies, and in 33 wastewater samples obtained at five health care institutions (HCIs), seven municipal wastewater treatment plants (mWWTPs), and an airport WWTP. Overall, 846 surface water and 313 wastewater *E*. *coli* isolates were analysed with respect to susceptibility to eight antimicrobials (representing seven different classes): ampicillin, cefotaxime, tetracycline, ciprofloxacin, streptomycin, sulfamethoxazole, trimethoprim, and chloramphenicol.

**Results:**

Among surface water isolates, 26% were resistant to at least one class of antimicrobials, and 11% were multidrug-resistant (MDR). In wastewater, the proportions of AMR/MDR *E*. *coli* were 76%/62% at HCIs, 69%/19% at the airport WWTP, and 37%/27% and 31%/20% in mWWTP influents and effluents, respectively. Median concentrations of MDR *E*. *coli* were 2.2×10^2^, 4.0×10^4^, 1.8×10^7^, and 4.1×10^7^ cfu/l in surface water, WWTP effluents, WWTP influents and HCI wastewater, respectively. The different resistance types occurred with similar frequencies among *E*. *coli* from surface water and *E*. *coli* from municipal wastewater. By contrast, among *E*. *coli* from HCI wastewater, resistance to cefotaxime and resistance to ciprofloxacin were significantly overrepresented compared to *E*. *coli* from municipal wastewater and surface water. Most cefotaxime-resistant *E*. *coli*isolates produced ESBL. In two of the mWWTP, ESBL-producing variants were detected that were identical with respect to phylogenetic group, sequence type, AMR-profile, and ESBL-genotype to variants from HCI wastewater discharged onto the same sewer and sampled on the same day (A_1_/ST23/CTX-M-1, B2_3_/ST131/CTX-M-15, D_2_/ST405/CTX-M-15).

**Conclusion:**

In conclusion, our data show that MDR *E*. *coli* are omnipresent in Dutch surface water, and indicate that municipal wastewater significantly contributes to this occurrence.

## Introduction

The use of antimicrobials in human and animal health care has resulted in the widespread prevalence of antimicrobial resistant (AMR) bacteria not only in humans and animals, but also in the environment, e.g. in surface water and soil [1–4]. As a consequence, the probability of getting exposed to AMR bacteria outside a health care setting has increased. For instance, people may get exposed through the preparation and consumption of contaminated meat products [5–7], vegetables, herbs and fruits [8–14], or contact with and ingestion of contaminated surface water, for instance during recreational activities [15,16]. Dissemination of bacteria with acquired resistance to antimicrobials may represent both direct and indirect risks to human health. The direct risk entails exposure to AMR pathogens, resulting in hard to treat infections. Indirect risks are associated with the exposure to relatively harmless AMR bacteria, such as commensal bacteria that are able to colonize gut, skin or mucosa, resulting in asymptomatic carriage of AMR bacteria. The public health risks associated with asymptomatic carriage comprise transfer of resistance genes between commensals and pathogens, transfer of AMR commensals (that are often opportunistic pathogens) to people who are more vulnerable to infection (e.g. the elderly, immunocompromised individuals and individuals with underlying disease), or asymptomatic carriers entering a stage of increased vulnerability themselves (e.g. hospitalization).

The threat AMR poses on global public health necessitates mitigation of dissemination of AMR bacteria, and therefore the identification of possible dissemination routes. One of the routes that should be considered, in particularly with respect to dissemination of faecal AMR bacteria, is surface water. Faecal bacteria, among which AMR variants, are emitted to the aquatic environment with human sewage and animal faeces. In the Netherlands, AMR bacteria are highly prevalent among livestock, in particular broilers, veal calves and slaughter pigs [17]. This high prevalence is related to years of massive use of antimicrobials in animal husbandry [17], a practice which has been curbed five years ago as a result of a covenant between multiple parties in the animal husbandry sector. Notwithstanding the concomitant decrease in antimicrobial use in Dutch livestock during the past couple of years, the prevalence of AMR gut bacteria is still very high. For example, for enterococci and *E*. *coli* prevalence figures of 50% to over 90% are reported for specific antimicrobial—animal combinations [17]. Contamination of surface water with faeces from livestock may occur through run-off of manure that is applied to the land for fertilization, or manure introduced unintentionally through run-off from farm premises or in the form of droppings of pasture and free-range animals. Faecal contamination of surface water through sewage occurs through the discharge of treated wastewater or the discharge of untreated sewage through sewage overflows during heavy rainfall. In the Netherlands, 352 wastewater treatment plants (WWTPs) are operational, the majority of which use biological treatment processes to reduce organic matter and nutrients. Only a small minority of these WWTPs use additional disinfection processes, such as UV, ozone treatment or membrane filtration to specifically remove microorganisms. During biological wastewater treatment without disinfection, concentrations of faecal bacteria are only partially reduced, e.g. for coliform bacteria in the order of 1–3 log_10_ units [18]. AMR bacteria and AMR genes are highly prevalent in sewage and sludge, and multiple studies have indicated that treated wastewater may indeed contribute to the contamination of surface water with AMR bacteria [19–29].

Surface water is an important source for drinking water production and is used for recreational activities and irrigation of crops. Through these uses, the human population may be exposed to AMR bacteria. Moreover, also animals (livestock, wild life) may be exposed to these bacteria, by drinking from or foraging in contaminated water. Over the past eight years, our research group has been engaged in multiple small-scale projects concerning the prevalence of AMR *E*. *coli* in surface water and wastewater, under the authority of different governmental bodies and ministries. The main goal of the present study was to gain insight into the prevalence and concentrations (and variation therein) of AMR *E*. *coli* in Dutch surface water, by an integrated data analysis of results from these multiple small-scale studies, and to explore the possible role of wastewater as AMR contamination source of Dutch surface water.

## Materials and Methods

### Sample description

Between 2006 and 2013, 30 surface water locations were sampled as part of different small-scale research projects on the authority of the Ministry of Infrastructure and Environment. Sampling sites were situated in different parts of the country, and consisted of different types of waterbodies: rivers, canals, rivulets, lakes and the North Sea. Sites were sampled once or multiple times, resulting in 113 samples (Table 1). For purpose of analysis of proportions of AMR and multidrug resistant (MDR) *E*. *coli* (i.e. to minimize the introduction of inaccuracies due to relatively low numbers of isolates per sample) the surface waters were grouped based on sampling region and year of sampling (Table 1). Surface water samples (1 liter) were taken according to NEN-EN-ISO 19458 [30].

**Table 1:** Surface water samples.

Group	Year of sampling	No. samples^**a**^	No. locations^**a**^	Types of water (number of different water bodies)
A	2006	24	4	Rivers (3), rivulet (1)
B	2008/2009	13	1	River (1)
C	2008/2009	14	1	River (1)
D	2008/2009	13	1	River (1)
E	2012	9	3	Canals (2), lake (1)
F	2012	8	2	Lake (1; two different sites)
G	2012	12	4	Canals (3), North sea (1)
H	2012	9	3	River (1; two different sites), lake (1)
I	2013	11	11	Rivulets (11)

^a^A total of 113 samples were taken at 30 locations scattered over eight different regions (group A and group H contained different water bodies, but were located in the same regional area). For each group, the number of samples analysed per location is calculated by dividing the no. of samples by the no. of locations; this number also equals the number of different sampling dates per site.

Between 2009 and 2013, wastewater was sampled at multiple wastewater treatment plants (WWTP) and health care institutions (HCIs) (Table 2). Five HCIs were sampled: two nursing homes (HCI-1, HCI-2), one top-clinical regional hospital (HCI-3), one regional hospital (HCI-4) and one university hospital (HCI-5). All HCI wastewater samples consisted of untreated wastewater, which was, in all cases but one, discharged onto the public sewage system. The exception was HCI-3, where at the time of the second sampling (2010), an experimental tertiary WWTP was operational (which included membrane filtration). Although in this case both influent and effluent were sampled, no *E*. *coli* was obtained from the effluent sample, and discussed results concern untreated hospital wastewater only. Wastewater was sampled at the WWTP of an international airport (aWWTP), where wastewater is derived from passengers, aviation industry and airplanes, and at seven municipal WWTPs (mWWTP-1 to mWWTP-7). Overall, seven influent samples (one from the aWWTP, six from mWWTPs) and 19 effluent samples (one from the aWWTP and 18 from mWWTPs) were obtained (Table 2). At the aWWTP and four of the mWWTP, influent and effluent samples were obtained at the same date and time. Each of the WWTPs including the aWWTP applied mechanical and biological treatment processes only, and effluents were not disinfected. Nursing home HCI-2 and regional hospital HCI-4 both discharged onto the public sewage treated at mWWTP-2, and the 2011 samples from these locations were taken at the same day. The same holds true for University Hospital HCI-5 and mWWTP-3. The majority of wastewater samples consisted of 24h flow proportional samples collected by staff from WWTPs, water boards or HCIs, using automated systems. When 24h flow proportional samples were not available (both samples of HCI-3, influent from mWWTP-1, and one of four effluent samples of mWWTP-3), grab samples were taken. All samples were transported to the laboratory shortly after sampling, where they were stored at 5±3°C and analysed within 24 hours.

**Table 2:** Wastewater samples.

Type of wastewater	No. samples	Sampling location (year of sampling)
Nursing home	2	HCI-1 (2009), HCI-2 (2011)
Hospital	5	HCI-3(2009, 2011); HCI-4 (2010, 2011); HCI-5 (2011)
Airport WWTP influent	1	aWWTP (2010)
Airport WWTP effluent	1	aWWTP (2010)
Municipal WWTP influent	6	mWWTP-1 (2009), mWWTP-2 (2010, 2011), mWWTP-3 (2011), mWWTP-6 (2013), mWWTP-7 (2013)
Municipal WWTP effluent^a^	18	mWWTP-2 (2010, 2011), mWWTP-3 (2011, 2012^*^) mWWTP-4 (2012^*^), mWWTP-5 (2012^ǂ^), mWWTP-6 (2012^*^, 2013), mWWTP-7 (2013)

HCI = Health Care Institution, WWTP = Wastewater Treatment Plant

^a^Effluents were obtained three (^*^) or four (^ǂ^) times during 2012.

Underlined years indicate that influents and effluents from the same year and location were sampled at the same time-point.

### Isolation and enumeration of *E*. *coli*

From each sample, multiple volumes were filtered through 0.45 μm pore size membrane filters (Millipore, Amsterdam, the Netherlands). Because samples were analysed as part of different projects, the method used for isolation and enumeration of *E*. *coli* varied and entailed either the use of tryptone soya agar (TSA) and tryptone bile agar (TBA), as described in to ISO 9308–1 ‘Rapid test’ [31], or alternatively, tryptone bile x-glucuronide agar (TBX) in accordance with ISO 16649–2 [32]. In short, filters were incubated on TSA or TBX for 4–5 hours at 36±2°C, and subsequently transferred to TBA or maintained on TBX and incubated for 19–20 hours at 44±0.5°C. Presumptive *E*. *coli* identified using the TSA/TBA method (i.e. indole-positive), were additionally confirmed as *E*. *coli* by testing for ß-glucuronidase-activity on Brilliance *E*. *coli*/coliform agar (BECSA; Oxoid, Badhoevedorp, the Netherlands) or using API20E (Biomerieux). Beta-glucuronidase-positive colonies identified using TBX were additionally confirmed by testing for indole-activity using BBL Dry Slide (BD, Breda, The Netherlands). *E*. *coli* concentrations were based on the number of indol-positive (TSA/TBA) or ß-glucuronidase-positive (TBX) colonies and the fraction of these colonies that was confirmed to be *E*. *coli*. The concentrations were calculated using Mathematica software 9.0.1 (WolframResearch, Champaign, IL, USA).

### Analysis of antimicrobial resistance

Overall, 1,159 *E*. *coli* isolates (846 from surface water and 313 from wastewater) were obtained. These were screened for susceptibility to a panel of antimicrobials of human and veterinary clinical relevance, using broth micro dilution supplemented with Etests (Biomerieux, Boxtel). For broth micro dilution, the Sensititre SensiTouch system (TREK, MCS Diagnostics, Swalmen) was used, using custom made sensititre plates. Both methods were performed according to the manufacturers’ instructions and CLSI guidelines [33]. All isolates were screened for susceptibility to one or two antimicrobial representatives from seven classes of antimicrobials: ampicillin (penicillins), cefotaxime (3^rd^ generation cephalosporins), tetracycline (tetracyclines), ciprofloxacin (fluoroquinolones), streptomycin (aminoglycosides), sulfamethoxazole and trimethoprim (folate pathway inhibitors), and chloramphenicol (phenicols). Resistance was defined as having a minimal inhibitory concentration (MIC) above the ecological cut-off value available at the EUCAST website [34]. Multi-drug resistance was defined as resistance to 3 or more different classes of antimicrobials [35]. Proportions of AMR and MDR resistant *E*. *coli* variants were calculated at the sample group level rather than at individual sample level, to minimize inaccuracies in proportions introduced by analysis of low numbers of isolates (on average 8 isolates per sample, range 1 to 15). Concentrations of AMR and MDR *E*. *coli* were estimated by multiplying the observed *E*. *coli* concentration in a sample, with the proportion of AMR and MDR *E*. *coli* calculated for the corresponding sample group.

### Antimicrobial resistance occurrence frequencies

For each type of antimicrobial resistance, the occurrence frequency within the different *E*. *coli* populations (population being defined as a set of *E*. *coli* isolates from one type of water source, e.g. surface water, HCI wastewater etc.) was calculated. This was done by dividing the times resistance to a specific antimicrobial was observed in an *E*. *coli* population by the total number of resistances observed in the same population. The obtained values were additionally compared with occurrence frequencies observed among *E*. *coli* from slaughter pigs, veal calves and broilers. For these animals the required parameters, i.e. total number of resistances (including only the types of resistance investigated in the current study) and the times resistance to a specific antimicrobial was observed, were deduced from percentages resistant *E*. *coli* and total numbers of isolates tested, which were reported as part of Dutch surveillance studies performed during 2010 and 2011 [36].

### Isolation and enumeration of ESBL-producing *E*. *coli*

From 2010 onwards, samples were screened for the presence and numbers of ESBL-producing *E*.*coli*, using selective medium (49 of 113 surface water samples: groups E through I, and 30 of 33 wastewater samples). Either TBX supplemented with 1μg/ml of cefotaxime (CTX) or ChromID ESBL agar (Biomerieux) was used. Plates were incubated for 18–24 hours at 36±2°C, or for 4–5 hours at 36±2°C followed by 18–19 hours at 44±0.5°C or. Suspected ESBL-*E*. *coli* isolates (i.e. ß-glucuronidase-positive on both types of medium) were confirmed to be indole-positive using BBL Dry Slide (BD), and subsequently tested for ESBL-production by disk diffusion following CLSI guidelines [33], using Sensi-Discs (BD, Breda, the Netherlands). Zone diameters were determined for cefotaxime (30μg), cefotaxime (30μg) + clavulanic acid (10μg), ceftazidime (30μg), ceftazidime (30μg) + clavulanic acid (10μg), and cefoxitin (30 μg). ESBL-producing isolates were defined as strains resistant to cefotaxime (zone diameter ≤ 22 mm) and/or ceftazidime (zone diameter ≤ 17 mm), and an increase in zone diameter of ≥ 5 mm with the disks containing clavulanic acid [33]. Isolates without a significant effect of clavulanic acid and resistant to cefoxitin (zone diameter ≤ 14 mm) were considered AmpC-producing [37]. ESBL-producing *E*. *coli* concentrations were calculated from the numbers of ß-glucuronidase-positive colonies, and the fraction of isolates confirmed to be indole-positive and ESBL-producing. Concentrations were calculated using Mathematica software 9.0.1 (WolframResearch, Champaign, IL, USA).

### Characterization of wastewater ESBL-producing isolates

The set of wastewater samples included two small wastewater chains: one consisting of nursing home HCI-2, hospital HCI-4 and mWWTP-2, and one consisting of hospital HCI-5 and mWWTP-3. For ESBL-producing isolates from these samples, AMR profiles, ESBL-genes, phylogenetic groups and sequence types were determined in order to establish potential relationships between strains detected in different samples along the water chains. AMR profiles were determined for the same antimicrobials used for *E*. *coli*, and additionally for ceftazidime and nalidixic acid (using Etests). For ESBL-gene analysis, the presence of genes encoding CTX-M-group 1, CTX-M-group 2, and CTX-M-group 9 ESBL, and *bla*_OXA_-, *bla*_SHV_- and *bla*_TEM_-genes, was established by multiplex PCRs using primers, primer concentrations and amplification conditions described by Dalenne et al. [38]. PCR products of the expected size were treated with ExoSAP-IT (GE Healthcare, Hoevelaken, the Netherlands) and sequenced using the same primers used for PCR. Obtained sequences were compared with ESBL-gene sequences in the GenBank database and on the Lahey website (www.lahey.org/studies). Isolates were allotted to phylogenetic groups A, B1, B2 or D, using PCR targeted to the *chuA*, *yjaA* genes and TspE4.C2 DNA fragment, using primers described by Clermont et al. [39] at a concentration of 0.2μM. Amplification conditions were as follows: 5 min 95°C, followed by 35 cycles of 30 s 95°C, 30 s 62°C, 30 s 72°C, and a final elongation step of 10 min 72°C. Strains were sub-grouped in seven groups/subgroups: A_0_, A_1_, B1, B2_2_, B2_3_, D_1_, and D_2_, according to the definitions used by Escobar-Páramo et al. [40]. Isolates identical with respect to AMR profile, ESBL-genotype and phylogenetic group were further characterized using multi-locus sequence typing (MLST) as described by Wirth et al. [41]. Primer sequences were obtained from the *E*. *coli* MLST database website http://mlst.warwick.ac.uk/mlst and used in a concentration of 0.2μM. Amplification conditions were as follows: 5 min 95°C, followed by 35 cycles of 30s 95°C, 30s 60°C (*adk*, *icd*, *mdh*, *purA*, *recA*) or 30s 64°C (*fumC*, *gyrB*), 45s 72°C, and a final elongation step of 10 min 72°C. PCR-products of the expected size were treated with ExoSAP-IT (GE Healthcare) followed by sequencing using the same primers used for PCR. Obtained sequences were imported in the *E*. *coli* MLST database website to determine MLST types.

### Statistics

The independent sample Kruskal Wallis Test was used to compare *E*. *coli* populations from different water sources with respect to the average number of resistances per isolate. The Pearson Chi-Square test was used to compare the proportions of antimicrobial resistant *E*. *coli* (i.e. with respect to AMR, MDR, and individual resistance types), and antimicrobial resistance frequencies between different *E*. *coli* sources. The diversity of ESBL-producing isolates in different wastewater samples was calculated using the Simpson’s Index of Diversity (1-D), which was calculated from:

$$D=\frac{\sum_{i=1}^{N}n_{i}\left(n_{i}-1\right)}{N\left(N-1\right)}$$

where n_i_ represents the number of variants with the i^th^ pheno-/genotype, and N the total number of isolates.

## Results

### Prevalence of AMR resistant *E*. *coli* in surface waters and wastewaters

**Fig 1:**
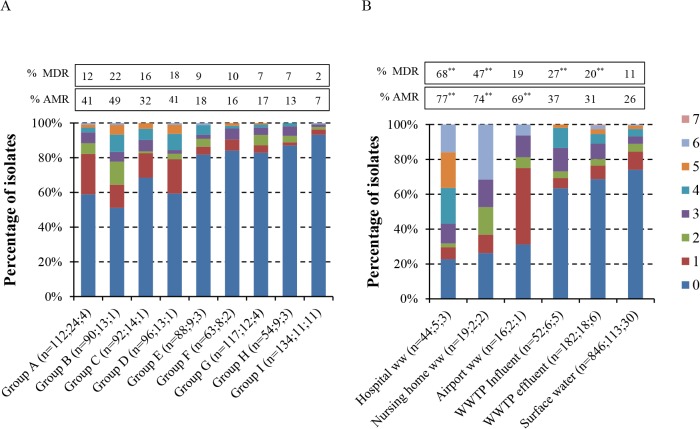
Resistant and multi-drug resistant *E*. *coli* in surface water (A) and wastewater (B). For each group of isolates from surface water (A) and wastewater (B), percentages of isolates resistant to 0, or 1 to 7 different antimicrobial classes are shown. On the x-axis, indicated between brackets (n = x;y;z) are the number of isolates (x), the number of samples (y), and the number of sample locations (z). MDR = multidrug resistant, i.e. resistant to three or more different classes of antimicrobials, AMR = antimicrobial resistant, i.e. resistant to at least one class of antimicrobials. For airport wastewater results from influent and effluent were combined because of small sample sizes. **P<0.01 relative to surface water values, Pearson Chi-Square Test.

The proportion of *E*. *coli* isolates resistant to at least one class of antimicrobials (AMR *E*. *coli*) varied from 7% to 49% between surface water groups (Fig 1A). The proportion of MDR *E*. *coli* varied from 2% to 22%. Overall, 26% of all 846 *E*. *coli* isolates from surface water were AMR, and 11% were MDR (Fig 1B). Among *E*. *coli* isolates originating from hospital wastewater the proportions of AMR (77%) and MDR (68%) *E*. *coli* were significantly higher compared to those from surface water (*P*<0.001, Pearson Chi square test). This was also the case for isolates from nursing home wastewater, with 74% and 47% of the *E*. *coli* being AMR and MDR, respectively (relative to surface water: *P*<0.0001, Pearson Chi Square test). In wastewater from municipal WWTPs, the proportions of AMR variants were slightly (and statistically not significant) higher than those in surface water: 37% and 31% in influents and effluents respectively, vs. 26% in surface water; differences with respect to the proportions of MDR variants were more pronounced: 27% and 20%, respectively, vs. 11% in surface water (*P* = 0.01, Pearson Chi Square Test; Fig 1B).

Compared to municipal wastewater, airport wastewater contained relatively high proportions of AMR *E*. *coli* (69%) but similar proportions of MDR *E*. *coli* (19%). In WWTP effluents, the proportions of AMR and MDR *E*. *coli* were slightly reduced relative to those in influents (AMR: 31% vs. 37%, MDR: 20% vs. 27%, respectively). Six of the effluent and influent samples were obtained at the same time at the same WWTP (once at the aWWTP and five times at mWWTP, see Table 2). When only these six pairs of effluent and influent samples were included for analysis, the difference in proportions of AMR and MDR *E*. *coli* between both types of wastewater appeared more distinct: respectively 30% (16/53) and 45% (23/51) of the *E*. *coli* isolates from matching effluent and influent were resistant to at least one antimicrobial, and 17% (9/53) and 27% (14/51) were MDR. Although these differences were not statistically significant (*P* = 0.1, Pearson Chi Square Test), these results may indicate a slightly higher decline rate for AMR *E*. *coli* than for susceptible *E*. *coli* during wastewater treatment.

### Detection frequencies of resistance types among *E*. *coli* from different water sources

In surface water and municipal wastewater, *E*. *coli* isolates resistant to sulfamethoxazole, trimethoprim, ampicillin, streptomycine or tetracycline (from here on indicated as ‘STAST’ antimicrobials) were more prevalent than isolates resistant to cefotaxime, ciprofloxacin or chloramphenicol (Table 3, left). In contrast, in HCI wastewater ciprofloxacin-resistant isolates were as common as isolates resistant to either of the ‘STAST’ antimicrobials, and also cefotaxime-resistant isolates were frequently detected (Table 3, left). For each individual antimicrobial, the proportion of resistant isolates was significantly higher among isolates from HCI wastewater compared to isolates from surface water (*P*-values from <0.001 to 0.007, Pearson Chi Square Test; Table 3, left). With the exception of the proportion of cefotaxime—resistant isolates, this was also true for isolates from mWWTP wastewater (*P*-values from <0.001 to 0.048, Pearson Chi Square Test; Table 3, left).

**Table 3:** AMR resistance and relative distribution of resistance types among *E*. *coli* from different water sources and livestock

	Percentages of *E*. coli isolates resistant to antimicrobial (A)			Occurrence of resistance (%) relative tototal no. of resistances (B)					
	Surface water (n_ec_ = 846)	mWWTP ww (n_ec_ = 234)	HCI ww (n_ec_ = 63)	Surface water (n_r_ = 596)	mWWTP ww (n_r_ = 265)	HCI ww (n_r_ = 224)	Broilers (n_r_ = 2342) ^b^	Slaughter pigs (n_r_ = 1556) ^b^	Veal calves(n_r_ = 1095) ^b^
**Sulfamethoxazole**	15.5	21.8*	55.6**	22.0	19.4	15.6*	16.3**	20.0	16.8**
**Trimethoprim**	9.6	17.1**	49.2**	13.6	15.2	13.8	14.1	17.6*	13.7
**Ampicillin**	12.5	21.8**	57.1**	17.8	19.4	16.1	17.3	12.6**	14.1*
**Streptomycin**	11.1	19.2**	47.6**	15.8	17.1	13.4	15.6	20.7*	17.0
**Tetracycline**	12.9	17.9*	50.8**	18.3	16.0	14.3	13.5**	23.8**	22.6*
**Cefotaxime**	1.7	1.3	33.3**	2.3	1.1	9.4**	3.2	0.48**	0.74**
**Ciprofloxacin**	5.0	8.5*	54.0**	7.0	7.5	15.2**	14.4**	0.44**	6.6
**Chloramphenicol**	2.2	4.7*	7.9**	3.2	4.2	2.2	5.6*	4.4	8.6**
***Total***	*70* ^*a*^	*113* ^*a*^	*356* ^*a*^	*100*	*100*	*100*	*100*	*100*	*100*

Indicated are (A) the percentages of *E*. *coli* with the indicated type of resistance, proportional to the total number of *E*. *coli* (n_ec_) obtained from the indicated water sources and (B) the proportion of the indicated types of resistance, relative to the total number of resistances (n_r_) in the respective *E*. *coli* populations.

**P<0.01 and

*P<0.05 relative to surface water values, Pearson Chi Square Test.

mWWTP = municipal wastewater treatment plant

ww = wastewater

HCI-health care institution.

^a^ The sum of all percentages does not add-up to the total percentage of AMR isolates because of the existence of multidrug resistant isolates.

^b^Calculated from the numbers of *E*. *coli* isolates and the percentages of *E*. *coli* isolates resistant to the antimicrobials under study reported in MARAN 2012 [34].

The average number of resistances per isolate was 0.7 (596/846), 1.1 (265/234) and 3.6 (224/63) for *E*. *coli* isolates from surface water, municipal wastewater and HCI wastewater, respectively (*P*<0.0001, Kruskal-Wallis Test). Irrespective of the water source, resistance to each of the ‘STAST’ antimicrobials contributed 13% to 22% of all resistances in different *E*. *coli* populations (Table 3, right). Overall, the antimicrobial resistance occurrence frequencies were very similar for surface water and municipal wastewater (Table 3, right). By contrast, resistance to cefotaxime (9.4% vs. 2.3%, *P*<0.001, Pearson Chi Square Test) and to a lesser extent ciprofloxacin (15% vs 7%, *P*<0.001, Pearson Chi Square Test), constituted a significantly larger proportion of observed resistances among *E*. *coli* from HCI wastewater compared to *E*. *coli* from surface water. Similarly, some types of resistance occurred more frequently among isolates from livestock compared to isolates from surface water (e.g. ciprofloxacin-, tetracycline-, and chloramphenicol resistance in broilers, slaughter pigs and veal calves, respectively) (Table 3, right). By contrast, some resistance types occurred more frequently among surface water isolates compared to livestock isolates (e.g. cefotaxime-resistance compared to slaughter pig/veal calve isolates, ciprofloxacin-resistance compared to slaughter pig isolates). Cefotaxime-resistance constituted a low proportion of all resistances in *E*. *coli* from veal calves (0.74%) and slaughter pigs (0.48%), yet a comparatively high proportion of all resistances in *E*. *coli* from broilers (3.2%), a value intermediate to that observed in surface water (2.3%) and HCI wastewater (9.4%).

### Prevalence of ESBL- and AmpC- producing *E*. *coli*

Eight surface water isolates (0.95%), obtained from seven of 113 (6.2%) surface water samples had combined cefotaxime and ampicillin resistance, which is indicative of ESBL- or AmpC-production. Five of these isolates (all from group B) were identified as ESBL-producers, and two (both from group I) were identified as AmpC-producers; one β-lactam-resistant isolate (from group A) has not been tested for ESBL-production. Twenty-five wastewater isolates from 9 of 33 wastewater samples (27%) were suspected ESBL- or AmpC-producers. Of these isolates, six were from nursing homes (32% of nursing home isolates), 15 from hospitals (34%), one from aWWTP (6.3%), and three from mWWTPs (1.3%). Twenty-two were confirmed to produce ESBL, and three produced AmpC.

Samples obtained after 2009 (surface water groups E through I: n = 49 and wastewater: n = 30) were also cultured on ESBL-selective media. Using selective media, ESBL-producing *E*. *coli* were detected in 55% of the surface water samples (in all five surface water groups) and in 100% of the wastewater samples. The characteristics of the ESBL-producing *E*. *coli* from most of these isolates (surface waters groups E, F, G and H, and effluents from mWWTP-3 through mWWTP-6 that were obtained in 2012) have been described elsewhere [19].

### ESBL-producing *E*. *coli* in wastewater chains

Among wastewater samples, two wastewater chains had been included: one consisting of nursing home HCI-2, regional hospital HCI-4 and mWWTP-2, and one consisting of university hospital HCI-5 and mWWTP-3. ESBL-producing isolates from these samples were characterized with respect to AMR profiles, ESBL-genes and phylogenetic groups (Table 4). In the nursing home (HCI-2) and the regional hospital (HCI-4), little to no diversity was observed in pheno/genotypes among ESBL isolates (Simpson’s index of diversity of 0.20 and 0.00 respectively). By contrast, in the university hospital HCI-5 and municipal wastewater, diversity was high (Simpson’s index of diversity of 0.93, 1.00 and 0.89 for HCI-5, WWTP-2 and WWTP-3 respectively). In wastewater from each of the three HCIs, one pheno/genotype was prevalent with (near) identical counterparts in wastewater from the connected mWWTP (1A, 1C and 2C, see Table 4). These variants constituted 100% and 12.5% (1A), 90% and 12.5% (1C) and 12.5% and 5.6% (2C) of the isolates from HCI and connected mWWTP respectively. To further confirm the similarity between 1A-, 1C-, and 2C-type variants from different origin, the sequence type was determined for a subset of each of these variants. The isolates were identified as A_1_/ST23/CTX-M-1, B2_3_/ST131/ CTX-M-15 and D_2_/ST405/CTX-M-15.

**Table 4:** Characteristics of ESBL-producing *E*. *coli* isolates from wastewater chains.

Sampling location	Variant ID	No. resistances	AMR profile*	ESBL	Extra β-lacamases	Phylo-genetic subgroup	No. of isolates (%)	ST (no. of isolates tested)
*Wastewater chain 1*								
HCI-4 (n = 9)	**1A**	**9**	**AmCxCzTeStCiNaSuTr**	**CTX-M-1**	**TEM-1**	**A**_**1**_	**9 (100%)**	**ST23 (3)**
HCI-2 (n = 10)	1B	8	AmCxCzTeCiNaSuTr	CTX-M-1	TEM-1	B1	1 (10%)	n.t.
	**1C**	**9**	**AmCxCzTeStCiNaSuTr**	**CTX-M-15**	**OXA-1**	**B2**_**3**_	**9 (90%)**	**ST131 (3)**
mWWTP-2	1D	7	AmCxCzTeStSuTr	CTX-M-1	TEM-1	A_1_	1 (50%)	n.t.
Influent (n = 2)	**1C**	**9**	**AmCxCzTeStCiNaSuTr**	**CTX-M-15**	**-**	**B2**_**3**_	**1 (50%)**	**ST131 (1)**
mWWTP-2	1E	5	AmCxCzCiNa	CTX-M-14	-	A_1_	1 (16.7%)	n.t.
Effluent (n = 6)	1F	5	AmCxCzTeSu	CTX-M-1	-	B1	1 (16.7%)	n.t.
	1G	7	AmCxCzStCiNaTr	CTX-M-1	-	A_1_	1 (16.7%)	n.t.
	1H	7	AmCxCzTeStSuTr	CTX-M-1	-	A_1_	1 (16.7%)	n.t.
	1I	7	AmCxCzTeStSuTr	CTX-M-1	-	B1	1 (16.7%)	n.t.
	**1A**	**9**	**AmCxCzTeStCiNaSuTr**	**CTX-M-1**	**TEM-1**	**A**_**1**_	**1 (16.7%)**	**ST23 (1)**
*Wastewater chain 2*								
HCI-5 (n = 8)	2A	5	AmCxCzCiNa	CTX-M-15	TEM-1	B2_3_	2 (25%)	n.t.
	2B	6	AmCxCzStCiSu	SHV-12	-	D_2_	1 (12.5%)	n.t.
	**2C**	**6**	**AmCxCzTeCiNa**	**CTX-M-15**	**OXA-1**	**D**_**2**_	**1 (12.5%)**	**ST405 (1)**
	2D	7	AmCxCzTeStSuTr	CTX-M-14	TEM-1	D_2_	1 (12.5%)	n.t.
	2E	9	AmCxCzTeStCiNaSuTr	CTX-M-15	TEM-1, OXA-1	B2_3_	2 (25%)	n.t.
	2F	9	AmCxCzTeStCiNaSuTr	CTX-M-15, SHV-12	TEM-1, OXA-1	A_0_	1 (12/5%)	n.t.
mWWTP-3	2G	4	AmCxCzCh	CTX-M-14	-	B2_3_	1 (12.5%)	n.t.
Influent (n = 8)	**2C**	**6**	**AmCxCzTeCiNa**	**CTX-M-15**	**OXA-1**	**D**_**2**_	**1 (12.5%)**	**ST405 (1)**
	2H	8	AmCxCzTeCiNaSuTr	CTX-M-15	-	A_1_	1 (12.5%)	n.t.
	2I	9	AmCxCzTeStCiNaSuTr	CTX-M-27	-	B2_3_	1 (12.5%)	n.t.
	2J	10	AmCxCzTeStCiNaSuTrCh	CTX-M-1	TEM-1	A_0_	4 (50%)	n.t.
mWWTP-3	2K	5	AmCxCzSuTr	CTX-M-1	-	A_0_	2 (20%)	n.t.
Effluent (n = 10)	2L	5	AmCxCzSuTr	CTX-M-1	-	B2_2_	1 (10%)	n.t.
	2M	6	AmCxCzStTrSuTr	CTX-M-1	-	B2_2_	1 (10%)	n.t.
	2N	7	AmCxCzCiNaSuTr	CTX-M-15	-	B2_3_	1 (10%)	n.t.
	2O	7	AmCxCzStCiSuTr	CTX-M-9, SHV-12	-	D_2_	1 (10%)	n.t.
	2P	8	AmCxCzTeStCiSuTr	CTX-M-15	TEM-1	D_1_	1 (10%)	n.t.
	2I	9	AmCxCzTeStCiNaSuTr	CTX-M-27	-	B2_3_	1 (10%)	n.t.
	2J	10	AmCxCzTeStCiNaSuTrCh	CTX-M-1	TEM-1	A_0_	2 (20%)	n.t.

* Am = ampicillin

Cx = cefotaxime

Cz = ceftazidime

Te = tetracyclin

St = streptomycin

Ci = ciprofloxacine

Na = nalidixic acid

Su = sulfamethoxazole

Tr = trimethoprim

Ch = chloramphenicol.

MICs were determined with Etests (Cz, Na, Su, Ch) and microbroth dilution (remainder of antimicrobials), resistance defined as MIC greater than or equal to the epidemiological cut-off (EUCAST).

Indicated in bold are pheno-/genotypes present in HCI and municipal wastewater.

### Concentrations of AMR, MDR and ESBL-producing *E*. *coli* in wastewater and surface water

**Fig 2:**
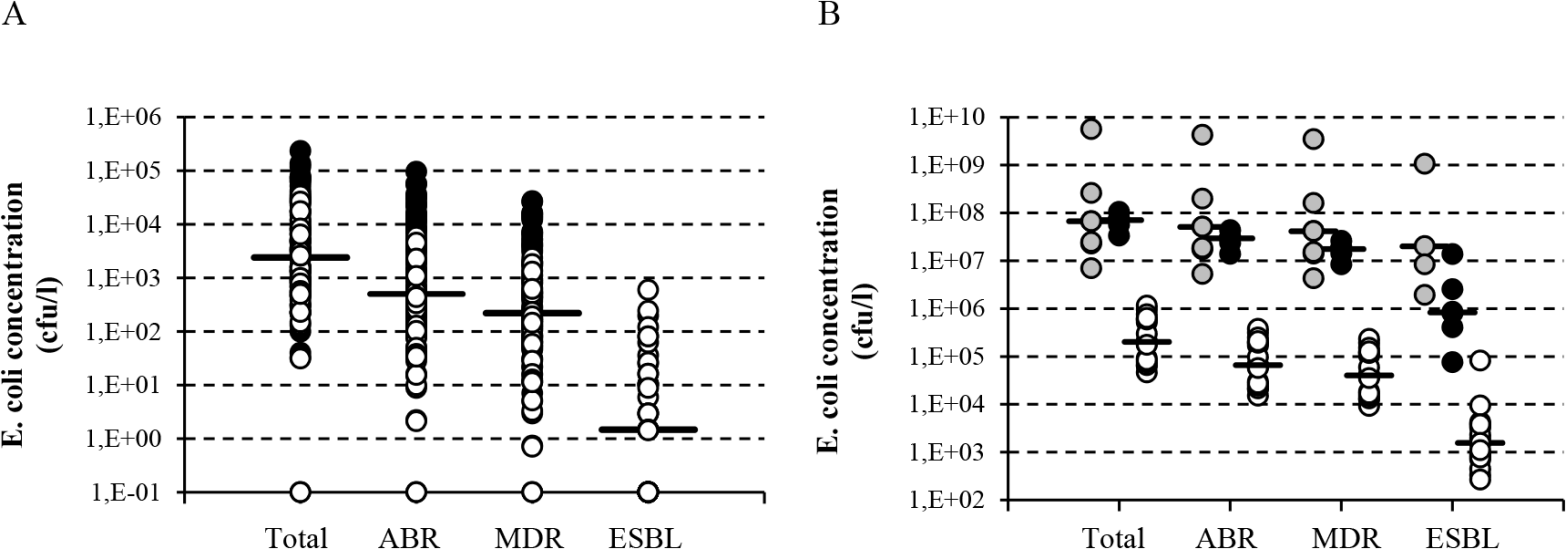
Bacterial concentrations in surface waters (A) and waste water (B). For purpose of comparison with concentrations of ESBL-producing *E*. *coli* which were only determined in samples from 2010 onwards, in (A), samples taken prior to 2010 are indicated in black, and samples taken from 2010 onwards are indicated in white. In (B), grey symbols indicate HCI wastewater, black symbols indicate WWTP influents, and white symbols indicate WWTP effluents. Horizontal bars indicate median values. Samples with concentrations below the detection limit (*E*. *coli*: <10 cfu/ml, ESBL-*E*. *coli*:.<1.5 cfu/ml) are represented by symbols on the x-axis.

The median *E*. *coli* concentration in surface water was 2.4×10^3^ cfu/l (range <10–2.3×10^5^, Fig 2A). Based on these concentrations, the estimated median concentrations of AMR and MDR *E*. *coli* were 5.0×10^2^ (range <0.67–9.5×10^4^) and 2.2×10^2^ (range <0.22–2.7×10^4^) cfu/l, respectively. In the subset of samples where ESBL-producing *E*. *coli* were enumerated, total-, AMR-, MDR-, and ESBL-producing *E*. *coli* concentrations were, 9.9×10^2^, 1.5×10^2^, 88, and 1.5 (range <1.5–5.9×10^2^) cfu/l, respectively. Wastewater from HCIs and WWTP influents contained median *E*. *coli* concentrations of 6.7×10^7^ cfu/l and 7.2×10^7^ cfu/l respectively, while in WWTP effluents concentrations were approximately 2-log_10_ lower (Fig 2B). Estimated median concentrations of AMR and MDR *E*. *coli* were in the same order of magnitude, with a maximum difference of 0.5-log_10_ units relative to the median *E*. *coli* concentration observed in WWTP effluents. The median ESBL-concentrations were 2.0×10^7^, 8.2×10^5^, 1.5×10^3^ in HCI, WWTP influents and WWTP effluents respectively (Fig 2B).

## Discussion

Given the observed prevalence of AMR *E*. *coli* in surface water, transmission to humans through surface water contact is a realistic scenario. The chance of transmission will amongst others, depend on the function of the contaminated water body, e.g. whether it is used for recreational activities or irrigation. Water-borne transmission has been demonstrated to be a relevant route of transmission for faecal bacterial species, including *Campylobacter*, *Salmonella* and *E*. *coli* [16,42–45]. Additional studies have described outbreaks of infections with Enterobacteriaceae associated with consumption of fresh produce [46], which may take-up these bacteria via irrigation water [47]. These examples confirm the contribution of water in dissemination of gut bacteria to humans. This will also hold true for AMR variants of these species. To establish risks of human exposure quantitatively, the framework of quantitative microbial risk assessment could be used [48]. This approach was successfully applied for assessing exposure and infection risks of *Campylobacter* from food consumption [49] and of bacterial and other pathogens from consumption of drinking water and recreational water [50,51].

The prevalence of AMR *E*. *coli* varied between surface waters that differed with respect to region, type of water body, level of faecal contamination, and time of the year at sampling. Since the different surface waters had been investigated as part of different research projects, sampling was not set up to study factors that influence variation in prevalence of AMR bacteria in space and time, and data are therefore not suitable to draw conclusions in that regard. Presumably, prevalence may vary with the number of faecal contamination sources in the vicinity of sampling sites, the distance to these sources combined with hydrologic features of the receiving water body (e.g. volume, flow speed), the origin of the faeces (e.g. the proportions derived from human, live stock or wild life sources), and factors influencing when and how often emission takes place (continuously or variable with climate or season). For instance, run-off of manure from livestock is likely to be highest during the main manure application season (which in the Netherlands is from the end of February until early May), and dependent on weather conditions (e.g. more run-off during rainy days). Sewage overflows will contribute only during spells of heavy rainfall, while WWTPs are continuous sources of faecal bacteria.

With respect to the proportions of AMR and MDR variants, municipal wastewater contained intermediate numbers compared to surface water and HCI wastewater. Even though *E*. *coli* concentrations in mWWTP effluents were on average 2-log units higher than those in surface water, the proportions of AMR and MDR (including ESBL-producing) variants were, on average, not markedly different. Moreover, despite the fact that surface waters and wastewater investigated in the current study were largely unrelated in space and time, a near identical distribution of types of resistance among *E*. *coli* in municipal wastewater and surface water was noted. Especially when comparing less prevalent types of resistance, such as resistance to cefotaxime, ciprofloxacin, and chloramphenicol, the occurrence frequency of resistance types among *E*. *coli* from surface water and municipal wastewater were very similar, while both were markedly different for *E*. *coli* from HCI wastewater and livestock sources. These data suggest a major contribution of municipal wastewater to contamination of Dutch surface water at a country level. However, the relative contribution of animal and human sources presumably varies with region (urban vs. agricultural areas) as well as with season. Results from research performed by our group demonstrated elevated levels of ESBL-producing *E*. *coli* in surface water nearby broiler farms (H. Blaak et al. in preparation), indicating for instance, that also livestock farms must be taken into account. Future studies are needed to establish to what extent wastewater effluents contribute to the prevalence of AMR *E*. *coli* in Dutch surface water, proportional to that of other possible contamination sources. For instance, by comparing bacterial load and epidemiological links between isolates from different suspected contamination sources and in parallel sampled upstream and downstream surface water sites, during different seasons.

In HCI wastewater, approximately three quarters of *E*. *coli* were resistant to at least one antimicrobial, and half to two-third of all isolates were resistant to three or more different classes of antimicrobials. These proportions were significantly higher than those observed in incoming sewage at mWWTPs, despite the similar concentrations of total *E*. *coli* in HCI and untreated municipal wastewater. This is not surprising, considering that per head, the use of antimicrobials, and hence acquisition of resistance, is higher in hospitals compared to the community (in 2011 in the Netherlands the defined daily dose was 11 per 1000 inhabitant-days in the community versus 71 per 100 patient-days in hospitals) [52]. In this regard, hospitals and nursing homes can be viewed as ‘hotspots’ of AMR resistance. The observed skewed distributions of resistance to cefotaxime and ciprofloxacin in HCI wastewater relative to that in municipal wastewater presumably reflect the higher use of these antimicrobials in hospitals than in primary care [52]. At least part of the *E*. *coli* with such hospital-associated types of resistance detected in municipal sewage are likely to originate from HCIs. This was supported by the detection of ESBL-producing *E*. *coli* isolates identical with respect to AMR profile, ESBL-genes, phylogenetic group and sequence type, in HCI wastewater and wastewater from the downstream situated WWTPs. These variants were identified as A_1_/ST23/CTX-M-1, B2_3_/ST131/ CTX-M-15 and D_2_/ST405/CTX-M-15. CTX-M-15-producing *E*. *coli* ST131 and ST405 have been associated with urinary tract infections, are disseminated worldwide [53–55], and have previously been isolated from Dutch patients with bacteraemia [56].

Our data confirm results from previous studies performed in Ireland and Poland [25,57]. In the former study, ESBL-producing *E*. *coli* were demonstrated in hospital wastewater, sewage downstream of the hospital and during various stages of treatment in the receiving WWTP, even though based on AMR profiles and PFGE types, these isolates appeared not to be genetically related. In the latter study, an overlap in ESBL-genotypes was demonstrated in hospital wastewater and municipal wastewater, although no further tests were performed to establish the relationship between isolates from hospital and municipal wastewater.

## Conclusions

Multidrug resistant *E*. *coli*, among which ESBL-producing variants, were frequently detected in different types of surface waters located in different regions in the Netherlands that were sampled between 2006 and 2013. Our data indicate that municipal wastewater, amongst others containing wastewater from the community and from HCIs, significantly contributes to the presence of antimicrobial-resistant *E*. *coli* in surface water. The omnipresence of AMR *E*. *coli* in surface water substantiates the potential role of surface water in dissemination of AMR faecal bacteria. Given the serious threat posed by multidrug resistant bacteria on public health, timely and rigorous actions are needed to limit their spread. Prevention of contamination of the aquatic environment should be considered as one of the tools to reach that goal. Future research needs to be aimed at establishing both the exposure risks associated with surface waters specifically used for recreational activities and irrigation, and the relative contributions of different types of contamination sources and factors influencing variation in the prevalence of AMR bacteria in surface water. This knowledge can then be used to predict the effect of interventions, e.g. disinfection of HCI wastewater or mWWTP effluents, or manure treatment processes, on the level of surface water contamination and risks of human exposure.
